# *In vitro* versus cryo-induced capacitation of bovine spermatozoa, part 1: Structural, functional, and oxidative similarities and differences

**DOI:** 10.1371/journal.pone.0276683

**Published:** 2022-10-21

**Authors:** Filip Benko, Abdollah Mohammadi-Sangcheshmeh, Michal Ďuračka, Norbert Lukáč, Eva Tvrdá

**Affiliations:** 1 Faculty of Biotechnology and Food Sciences, Institute of Applied Biology, Slovak University of Agriculture, Nitra, Slovak Republic; 2 Department of Animal and Poultry Science, College of Aburaihan, University of Tehran, Pakdasht, Tehran, Iran; University Hospital of Münster, GERMANY

## Abstract

Low temperatures during cryopreservation activate a cascade of changes, which may lead into irreversible damage and reduction of the fertilization potential, including the process of premature capacitation. The aim of our study was to evaluate the range of cell damage following the cryopreservation process and possible activation of cryocapacitation in bovine spermatozoa. For the experiments semen samples were obtained from 30 sexually mature Holstein bulls. Within the analysed parameters, we focused on the functional activity, structural integrity, capacitation status and oxidative profile. The samples were divided into three experimental groups, control (CTRL), *in vitro* capacitated (CAP) and cryopreserved (CRYO). Based on the collected data, there was a significant decrease in the sperm motility, mitochondrial membrane potential and concentration of cyclic adenosine monophosphate in the CRYO group when compared to CAP and CTRL (P<0.0001). A significant decrease (P<0.01; P<0.0001) in the membrane and acrosome integrity as well as DNA fragmentation index and a significant increase (P<0.0001) of necrotic cells were observed in the CRYO group. Following capacitation, a significant increase (P<0.01; P<0.0001) was recorded in the number of cells which underwent the acrosome reaction in the CRYO group against CAP and CTRL. Changes in the oxidative profile of the CRYO group indicates an increase (P<0.0001) in the reactive oxygen species generation, except for the superoxide radical, which was significantly higher (P<0.0001; P<0.001) in the CAP group in comparison with CRYO and CTRL. In summary, premature capacitation may be considered a consequence of cryopreservation and the assessed parameters could serve as physical markers of cryogenic damage to bovine spermatozoa in the future.

## Introduction

**Fig 1:**
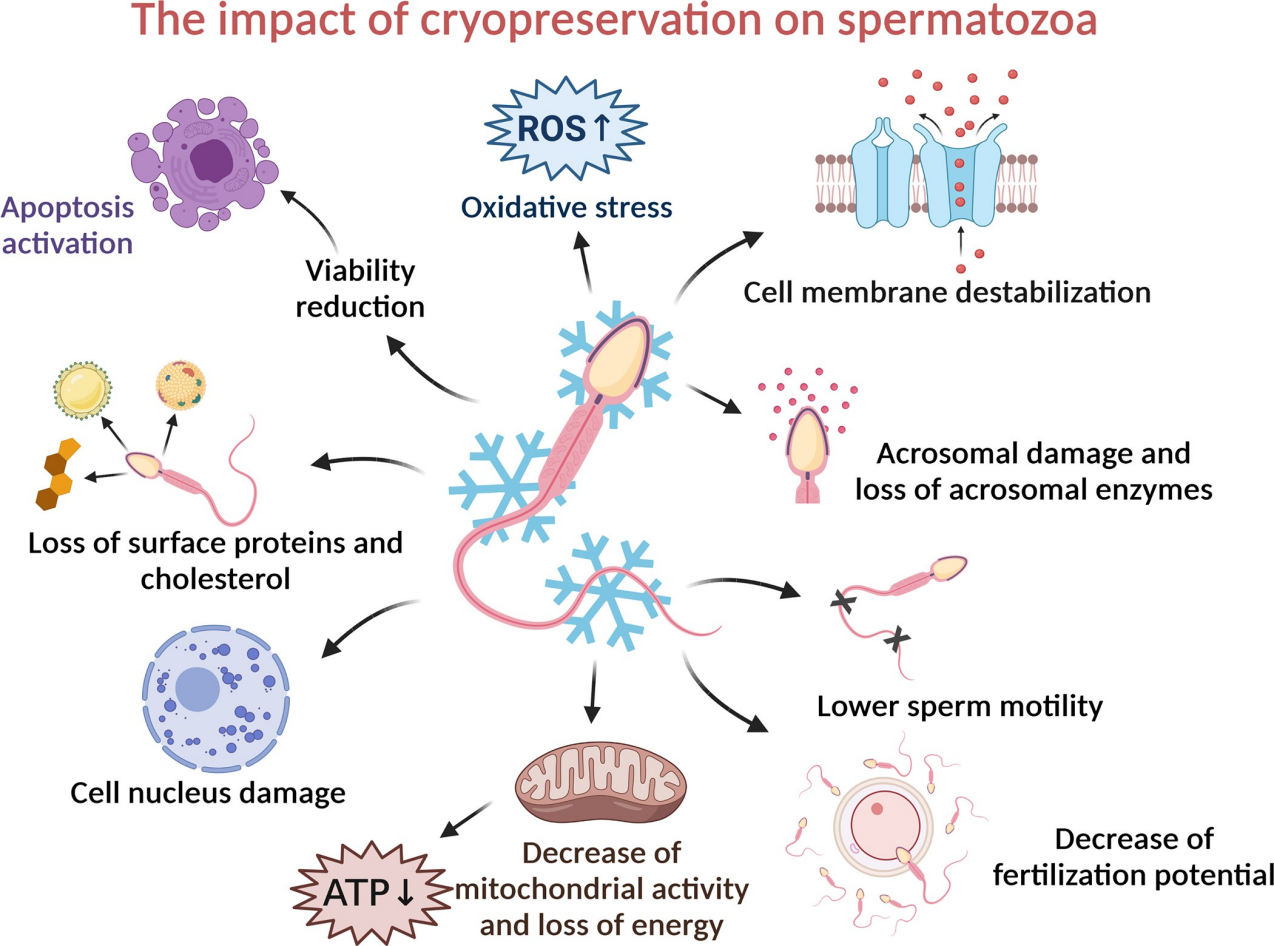
Consequences of cryodamage on spermatozoa including structural, functional, and molecular changes. Created with BioRender.com.

Globally, the cryopreservation of bovine spermatozoa has a positive impact on the livestock production. Through cryobiotechnologies and artificial insemination, it is possible to use the genetic material of valuable bulls and inseminate a larger number of cows, which increases the effectivity of cattle production. Despite that cryopreservation of bovine semen is technologically well managed, the viability of sperm cells after thawing decreases notably when compared to a native sample. During the freezing process, male gametes are exposed to physiological and structural changes due to heat shock, osmotic imbalance, and formation of intracellular ice crystals. The cryopreservation process may also negatively affect the plasma membrane, cytoskeleton, motion apparatus and destabilize the cell nucleus. Excessive mitochondrial activity induces the formation of reactive oxygen species (ROS) and a subsequent development of oxidative stress (Fig 1). In addition, differences in the shape, composition of the phospholipid layer of the sperm membranes and susceptibility to thermal shock have been reported among species. Bull, ram, stallion, and boar spermatozoa have been identified as the most cryosensitive in contrast to rabbit or human male gametes. Highly resistant spermatozoa are characterized by a higher level of polyunsaturated fatty acids and concentration of cholesterol in the cell membrane [1–3]. Nowadays, premature capacitation is considered to be one of the most pronounced contraindications of cryodamage that is involved in a reduction of viability, quality, and fertilization potential of post-thaw spermatozoa. In comparison to physiological capacitation, cryocapacitation is caused by the presence of low temperatures during the freezing and post-thawing process. Sperm cryopreservation is often associated with cell membrane destabilization, the loss of surface proteins, a decreased motility, mitochondrial activity, and an increased ROS generation. Cryocapacitation leads to membrane reorganization, accompanied with the loss of polyunsaturated fatty acids and cholesterol, which play important roles in maintaining a natural fluidity and integrity of the cell membrane [4,5].

Naturally, capacitation starts immediately following ejaculation into the urogenital tract of females. Mammalian sperm cells must undergo a series of biochemical and morphological modifications before acrosome reaction, which include an increase of HCO_3_^-^ (bicarbonate) concentration, removal of cholesterol from the plasma membrane, an increased membrane fluidity, intracellular pH and Ca^2+^ concentration, activation of PKA (protein kinase A), CatSper communication channels and cAMP/PKA pathways accompanied by a hyperactivated motility, which is one of the specific signs of capacitated spermatozoa [6–9].

The main issue of cryopreservation lies in that spermatozoa start capacitating right after thawing, which leads into a massive loss of energy needed for a successful fertilization. The process of cold-induced capacitation of spermatozoa is still poorly understood from a molecular perspective. Hence, the objective of our study was to unravel a spectrum of alterations to selected qualitative parameters of bovine spermatozoa caused by low temperatures and to discuss potential similarities with a natural capacitation process on different levels. Moreover, we also strived to quantify the levels of selected subclasses of free radicals involved in a physiological and/or premature capacitation and hence to evaluate the oxidative profile of *in vitro* capacitated and cryopreserved bovine spermatozoa.

## Materials and methods

### Biological material and *in vitro* cultivation of bovine spermatozoa

Ejaculates (n = 30) were obtained from sexually mature and healthy Holstein bulls using an artificial vagina at local farm (Slovenské biologické služby, a.s., Nitra). All samples were subjected to a primary motility analysis (min. 80% of motile sperm cells). Immediately, the samples were stored in a transport container and delivered to the laboratory for further processing. Each sample was divided into three parts; the first fraction was incubated in physiological saline solution (IMUNA PHARM, a. s., Šarišské Michaľany, Slovakia) in a ratio of 1:40 as the control. The second one was diluted (1:40) and incubated with pre-warmed capacitation medium [10] consisting of sodium chloride, potassium chloride, sodium bicarbonate, sodium phosphate monobasic, HEPES, magnesium and calcium chloride, sodium D-lactate and phenol red for the induction of capacitation under *in vitro* conditions. The data summarizing the volume of diluents and final concentration of spermatozoa in each sample are available in S1 Table in S1 File. The incubation time for both groups was 30 min. at 39°C and 5% CO_2_. After the incubation, the control as well as capacitated group was assessed by the CASA (computer-assisted sperm analysis) system for the evaluation of motility. If the capacitation was successful, capacitated spermatozoa were characterized by hyperactivated motility, which was visible under the microscope. The stability of the prepared capacitation medium was two weeks (4°C). The third part of each sample was frozen and stored in liquid nitrogen at -196°C for one month, then thawed and analysed. Each part of the samples was assessed separately, the individual groups being compared to understand the course and differences between a physiological capacitation and cryocapacitation. The cultivation temperature of 39°C was chosen based on natural temperature conditions of the urogenital tract of cows for the imitation of physiological conditions during the capacitation process.

### Cryopreservation of bovine spermatozoa

All semen samples (concentration 44x10^6^ spermatozoa/ml) were diluted with Triladyl containing 20% (w/v) egg yolk, citric acid, antibiotics, sucrose, Tris, glycerol, distilled water, and buffering agents. The data summarizing the volume of diluents and final concentration of spermatozoa in each sample are available in S1 Table in S1 File. Subsequently the diluted semen was transferred into 0.25 ml French straws using an automatic straw filler (Minitüb GmbH, Germany), cooled at 4°C for 2 hours and frozen according to the following scheme: -3°C/min. from +4°C to -10°C; -40°C/min. from -10°C to -100°C; -20°C/min. from -100°C to -140°C with a digital freezer (Digitcool Model 5300 ZB 250; IMV, France). Frozen samples were then transferred to liquid nitrogen at -196°C and stored for one month (Slovenské biologické služby, a.s., Nitra).

### Thawing and washing of samples

Cryopreserved samples were transported to the laboratory using a transport container filled with liquid nitrogen. Cryopreserved straws were thawed using a heating pad at 37°C for approximately 1–2 min. Thawed bovine spermatozoa were transferred to pre-prepared labelled 0.5 ml tubes and subjected to motility analysis by the CASA system. Subsequently, 500 μl PBS (phosphate buffer solution; Sigma-Aldrich, St. Louis, MO, USA) were added and the samples were centrifuged for 10 min./5000 RPM to remove the cryoprotectant residues. The washing process was repeated twice. The washed samples were subjected to further analyses.

### Sperm motility analysis

Motility of spermatozoa was assessed by computer-assisted sperm analysis (CASA; version 14.0 TOX IVOS II.; Hamilton-Thorne Biosciences, Beverly, MA, USA). Ten μl of each sample were pipetted into Makler’s counting chamber (depth 10 μm, 37°C; Sefi Medical Instruments, Haifa, Israel) and analysed. The results were evaluated using the Animal Motility program (Hamilton-Thorne, Biosciences, Beverly, MA, USA).

### Mitochondrial membrane potential

Mitochondrial membrane potential was quantified by the fluorescent dye JC-1 (5,5‘,6,6‘–tetrachloro-1,1‘,3,3‘-tetraethylbenzimidazolylcarbocyanineiodide), which is able to trespass the inner membrane of mitochondria and change their fluorescent properties. When the mitochondrial membranes are functional, they form polymers with JC-1, which is manifested by red fluorescence. In contrast, damaged mitochondrial membranes do not interact with the JC-1 dye which remains in a monomeric form, emitting a green fluorescent light [11,12].

### Membrane integrity

Evaluation of membrane integrity status was performed by triple fluorescent staining (carboxylfluorescein diacetate/CFDA, propidium iodide/PI and 4‘6-diamidine-2-phenylindole/DAPI; Sigma-Aldrich, St. Louis, MO, USA). Diluted samples of ejaculates were stained by adding 10 μl of each fluorescent dye and incubated for 15 min. at 37°C. This was followed by centrifugation at 5min./2100 RPM. The resulting supernatant was carefully removed, and samples were washed twice by adding 100 μl PBS. After the washing process, the supernatant was removed, the cells were diluted in 100 μl PBS and transferred to a prepared 96-well microplate and subsequently measured by the combined spectro-fluoro-luminometer Glomax Multi^+^ (Promega, Madison, WI, USA) at specific wavelengths [13].

### Acrosome integrity

The acrosome integrity of bovine spermatozoa was assessed using PNA lectin (Sigma-Aldrich, St. Louis, MO, USA) from *Arachis hypogaea*, which had the ability to bind with damaged sperm acrosome. We added 100 μl PNA and 10 μl DAPI into diluted samples, which were then incubated for 30 min. at 37°C. After incubation, the samples were transferred to a 96-well microplate and evaluated with the combined spectro-fluoro-luminometer Glomax Multi^+^ [13].

### Sperm DNA fragmentation

The level of DNA fragmentation was assessed with the SCD test (chromatin-dispersion test) and commercially available Halomax Kit (Halotech, Madrid, Spain). Spermatozoa were captured on a slide in an agarose matrix, followed by denaturation of DNA and removal of nuclear proteins. The amount of fragmented DNA was characterized by the presence or absence of a radiant border around the sperm head. The prepared slides were stained with Sybr Green (Sigma-Aldrich, St. Louis, MO, USA) and examined under a fluorescent microscope (Leica DMI6000 B; Wetzlar, Germany) with a 40x magnification, and a minimum of 300 visibly stained cells in each sample [14].

### Capacitation status

The capacitation process was assessed using fluorescent microscopy and chlortetracycline (CTC) assay (Sigma-Aldrich, St. Louis, MO, USA), which allows more detailed and reliable identification of capacitated spermatozoa. The principle of this method is to identify a specific pattern following absorption of the fluorescent dye by the sperm cells. Based on the coloration three patterns were recognized. The „F”pattern indicated a uniform fluorescence of the entire sperm head, which was characteristic for uncapacitated cells. Pattern „B”was visible as a narrow band of fluorescence in the postacrosome region of the sperm cell, which detected capacitated cells. The „AR”pattern indicated spermatozoa that underwent acrosome reaction, which was visible as a band of fluorescence in the sperm flagellum [15].

### Quantification of cyclic adenosine monophosphate (cAMP) concentration

For the determination of cAMP concentration, we used a commercial cAMP-Glo^TM^ kit (Promega, Madison, WI, USA), which works on the principle of protein kinase A stimulation with a decrease in adenosine triphosphate (ATP). These changes are characterized by a decrease in luminescent activity detected by luciferase, which was evaluated by the Glomax Multi^+^ combined spectro-fluoro-luminometer [16].

### Generation of reactive oxygen species (ROS)

The production of ROS was quantified by the chemiluminescence method, which employed luminol as a probe. The substance is able to interact with several types of ROS such as hydrogen peroxide, hydroxyl radical and superoxide anion. The values of chemilunescence were quantified with the combined spectro-fluoro-luminometer Glomax Multi^+^ [17].

### Production of superoxide anion

The quantity of intracellular superoxide radical was quantified using nitroblue-tetrazolium (NBT) assay. The principle of test consisted in conversion of yellow tetrazolium chloride (2,20 bis-(4-nitrophenyl)-5,50-diphenyl-3,30-(3,30-dimethoxy-4,40-diphenylene); Sigma Aldrich, USA) to blue formazan by the activation of superoxide radical. Optical density was evaluated spectrophotometrically with the Glomax plate reader [18].

### Production of hydrogen peroxide

A commercially available Amplex Red® Hydrogen Peroxide/Peroxidase Assay kit (Thermo Fisher, Waltham, MA, USA) was used for detection of hydrogen peroxide (H_2_O_2_) concentration. The principle is the detection of hydrogen peroxide or peroxidase by 10-acetyl-3-7-dihydroxyphenoxazine in red fluorescent oxidation product called resofurin. A working solution consisting of 10 mM Amplex Red® and 10 UI horseradish peroxidase was used to treat one million cells adjusted to 50 μL and the mixture was incubated at 37°C for 30 min. in the dark. The amount of resofurin was detected with the green fluorescent filter (Ex: 525 nm, Em: 580–640 nm) using the combined spectro-fluoro-luminometer Glomax Multi^+^.

### Production of hydroxyl radical

The extent of hydroxyl radical was quantified with the aminophenyl fluorescein fluorescent probe (APF; Thermo Fisher, Waltham, MA, USA). One million cells adjusted to 100 μL were stained with 2 μL of 100 μM APF staining solution and incubated at 37°C for 20 min. under dark conditions. Upon oxidation, APF exhibited bright green fluorescence, the intensity of which was analysed with the blue fluorescent filter (Ex: 490 nm, Em: 510–570 nm) using the combined the combined Glomax Multi^+^ spectro-fluoro-luminometer.

### Statistical analysis

The results were statistically processed by using GraphPad Prism program (version 6.0 for Windows, GraphPad Software incorporated, San Diego, California, USA, http://www.graphpad.com/). For the determination of significant differences between the control and experimental groups, One-way ANOVA together with Tukey’s range test were applied with the significance levels set at P<0.05; P<0.01; P<0.001; P<0.0001. Data in the graphs contain the average values for the individual groups ± SD (standard deviation).

### Ethics statement

The animals and sample collection were carefully handled in accordance with ethical guidelines as stated in the Slovak Animal Protection Regulation RD 377/12, which conforms to European Union Regulation 2010/63. Since semen collection is routinely performed at the Slovenské biologické služby a.s. breeding center, causing no harm or discomfort, a special Ethical Approval was not needed for this type of experiment.

## Results

The impact of cryodamage in relation to the course of capacitation was observed in a wide range of parameters such as functional activity, structural integrity, capacitation patterns and oxidative profile of bovine spermatozoa.

### Functional activity

**Fig 2:**
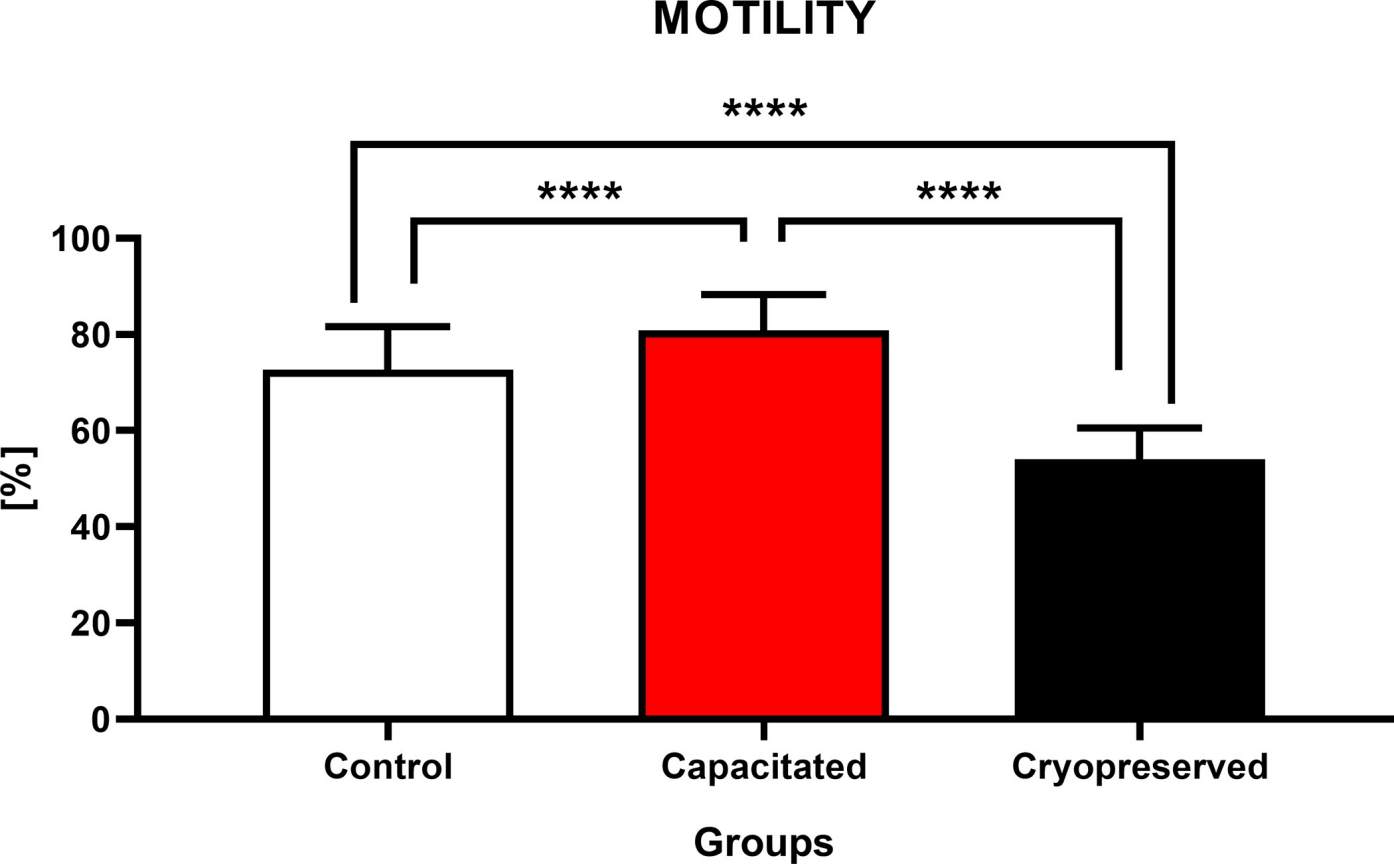
Motility of non-capacitated, *in vitro* capacitated and cryopreserved bovine spermatozoa. The values in the columns represent the proportion of motile spermatozoa (%) in each group (±SD). The results were obtained by comparing all groups with each other. The level of significance was set at *P<0.05; **P<0.01; ***P<0.001; ****P<0.0001.

**Motility of bovine spermatozoa.** The highest proportion of motile spermatozoa (80.9±7.4%) was recorded in the CAP group, which was significantly higher (P<0.0001) against CRYO and CTRL (72.6±9.0%). The lowest percentage (54.1±6.4%) of cells capable of moving was determined in the CRYO group, which was significantly lower (P<0.0001) when compared to CAP or CTRL (Fig 2).

#### Mitochondrial membrane potential

**Fig 3:**
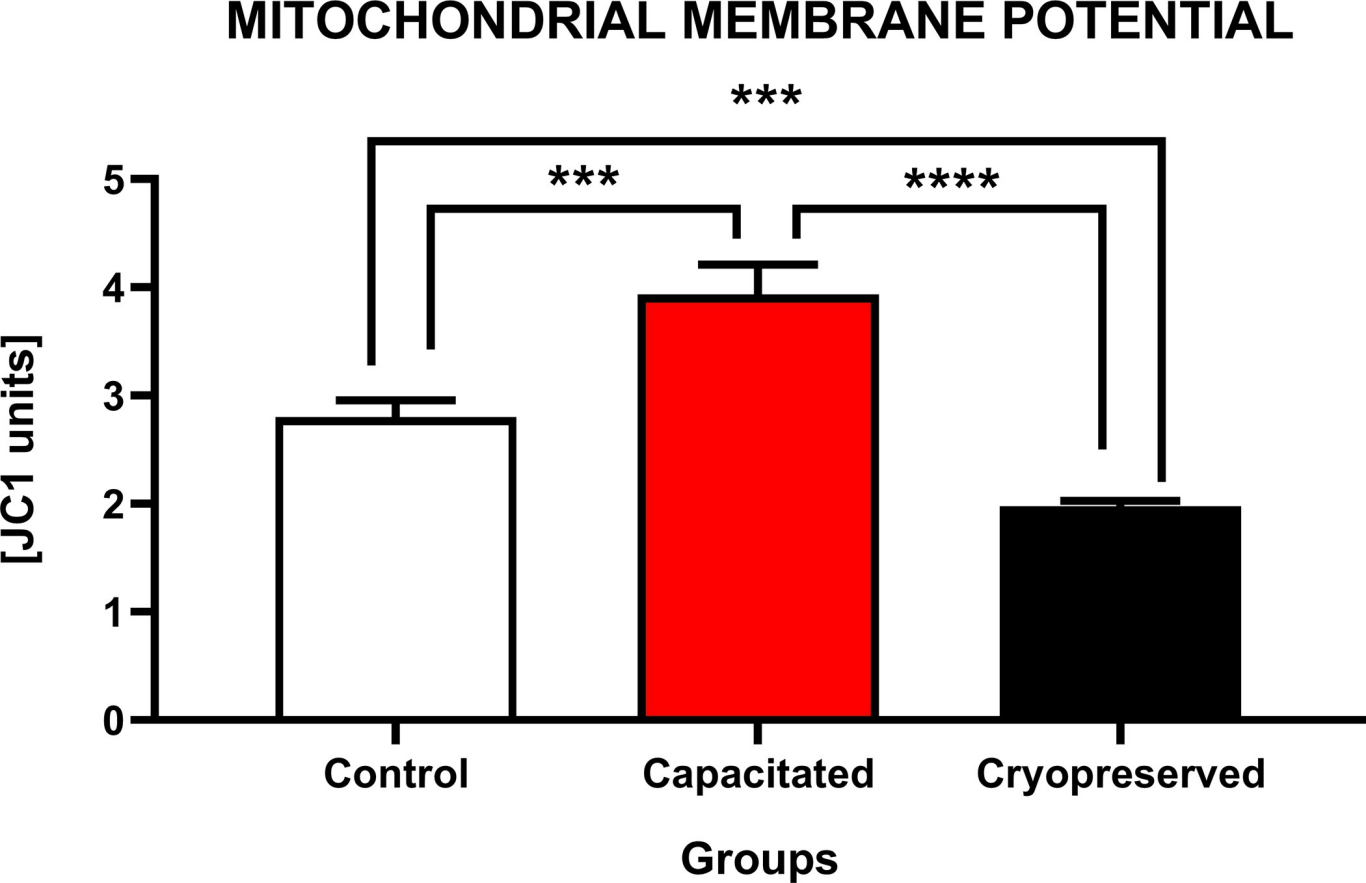
Mitochondrial membrane potential of of non-capacitated, *in vitro* capacitated and cryopreserved bovine spermatozoa. Each bar represents the values of mitochondrial activity (JC1 units) between experimental groups (±SD). The level of significance was set at *P<0.05; **P<0.01; ***P<0.001; ****P<0.0001.

Based on the results in Fig 3, the statistically highest mitochondrial activity was observed in the CAP against CRYO (P<0.0001) and CTRL (P<0.001). In contrast, in the CRYO group, a significant decrease (P<0.0001; P<0.001) in the mitochondrial activity was observed when compared to CAP and CTRL.

#### Concentration of cAMP

**Fig 4:**
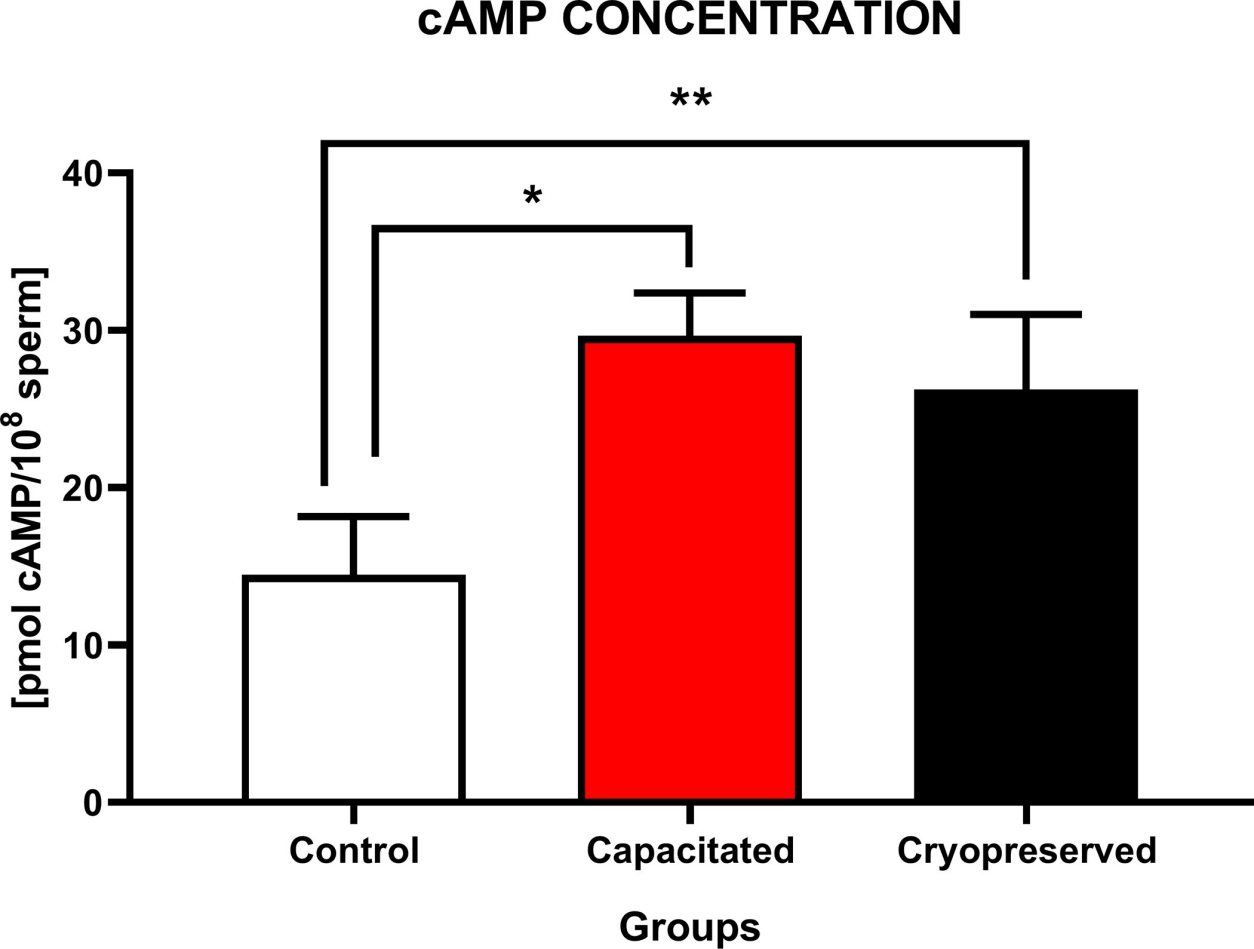
The concentration of cyclic adenosine monophosphate (cAMP) in non-capacitated, *in vitro* capacitated and cryopreserved bovine spermatozoa. The graph represented the concentration of cAMP (pmol cAMP/10^8^) between the groups (±SD). The level of significance was set at *P<0.05; **P<0.01; ***P<0.001; ****P<0.0001.

Data in Fig 4 show a significant increase (P<0.05) of cAMP concentration in CAP group when compared to CTRL. A statistically increased cAMP concentration (P<0.01) was also detected following comparison between CRYO and CTRL. No significant changes in cAMP concentration were observed between CAP and CRYO group.

### Structural integrity

#### Membrane integrity

**Fig 5:**
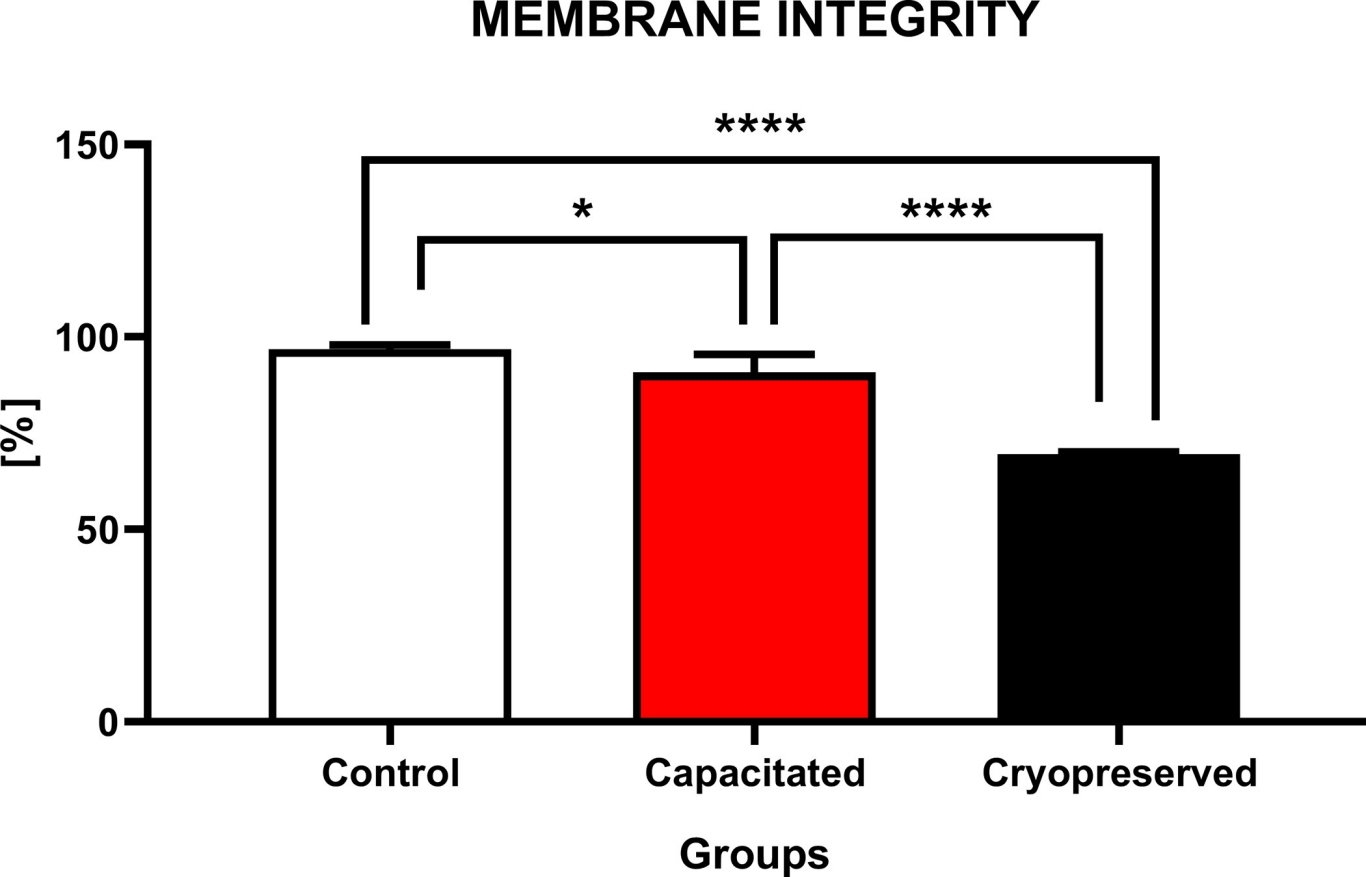
Membrane integrity of non-capacitated, *in vitro* capacitated and cryopreserved bovine spermatozoa. Each column showed the percentage of cells with intact cell membrane between the groups (±SD). The level of significance was set at *P<0.05; **P<0.01; ***P<0.001; ****P<0.0001.

The graph evaluating the membrane integrity (Fig 5) reveals that the highest percentage of cells with intact membranes was present in the CTRL (96.9±1.1%). There was a significant decline (P<0.05; P<0.0001) of membrane integrity in the CAP as well as CRYO group when compared to the CTRL. A significant loss of the membrane integrity (P<0.0001) was recorded also between CAP (90.8±4.6%) and CRYO group (69.5±2.6%), which was characterized by the lowest proportion of cells with intact membranes in this group.

#### Acrosome integrity

**Fig 6:**
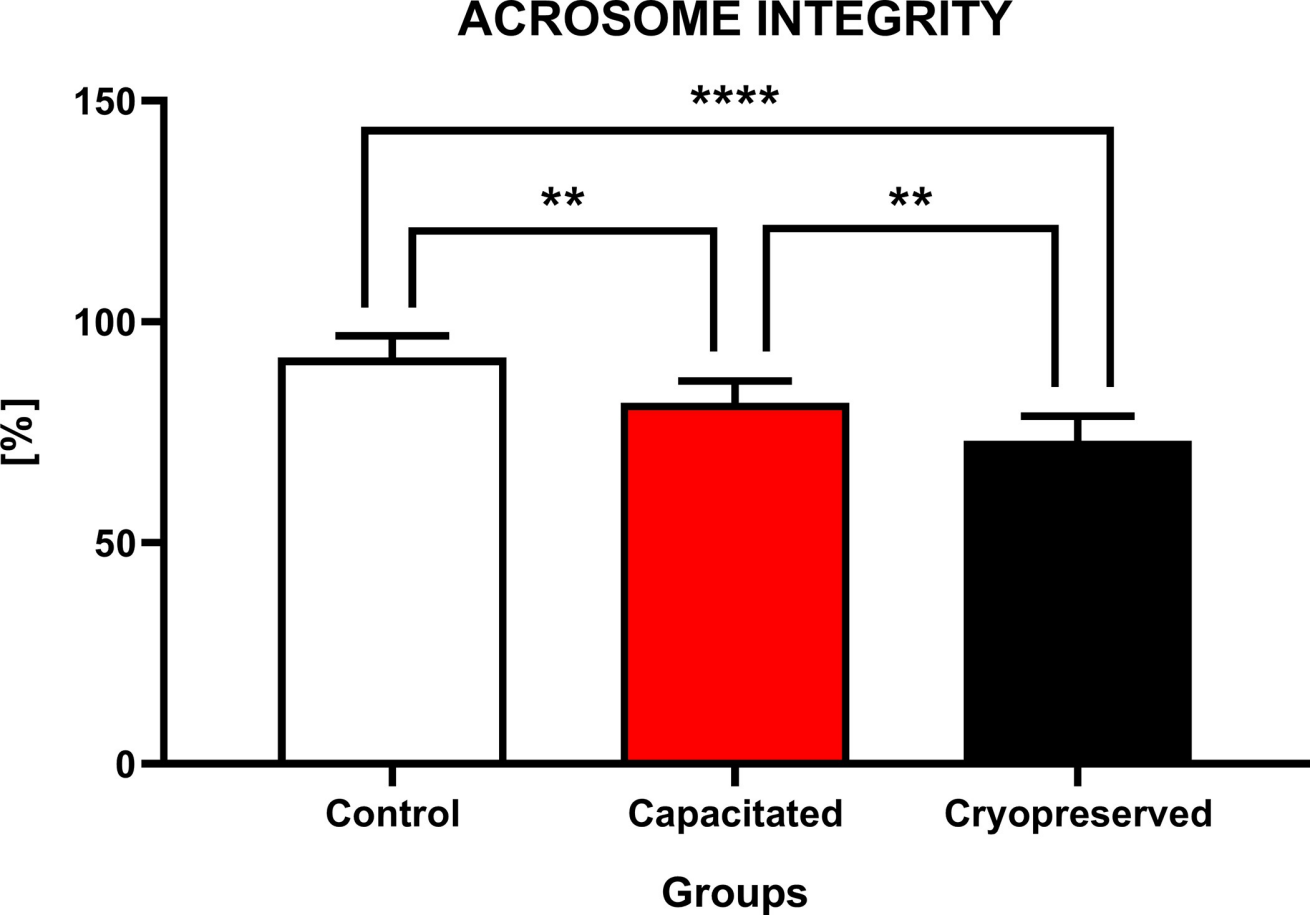
Acrosome integrity of non-capacitated, *in vitro* capacitated and cryopreserved bovine spermatozoa. Each bar represented the proportion of spermatozoa (%) with intact acrosome by comparing all groups with each other (±SD). The level of significance was set at *P<0.05; **P<0.01; ***P<0.001; ****P<0.0001.

Evaluation of the acrosome integrity indicates that the higher percentage of cells with intact acrosome was found in the CTRL (91.9±4.9%). In comparison to the CAP (81.7±4.9%) and CRYO group (73.1±5.6%), there was a continuous decline of cells with a functional acrosome. The graph also shows a significant decrease (P<0.01; P<0.0001) of acrosome integrity in the CAP and CRYO group against CTRL. However, significant changes (P<0.01) were also recorded between the CAP and CRYO group (Fig 6).

#### Cell necrosis

**Fig 7:**
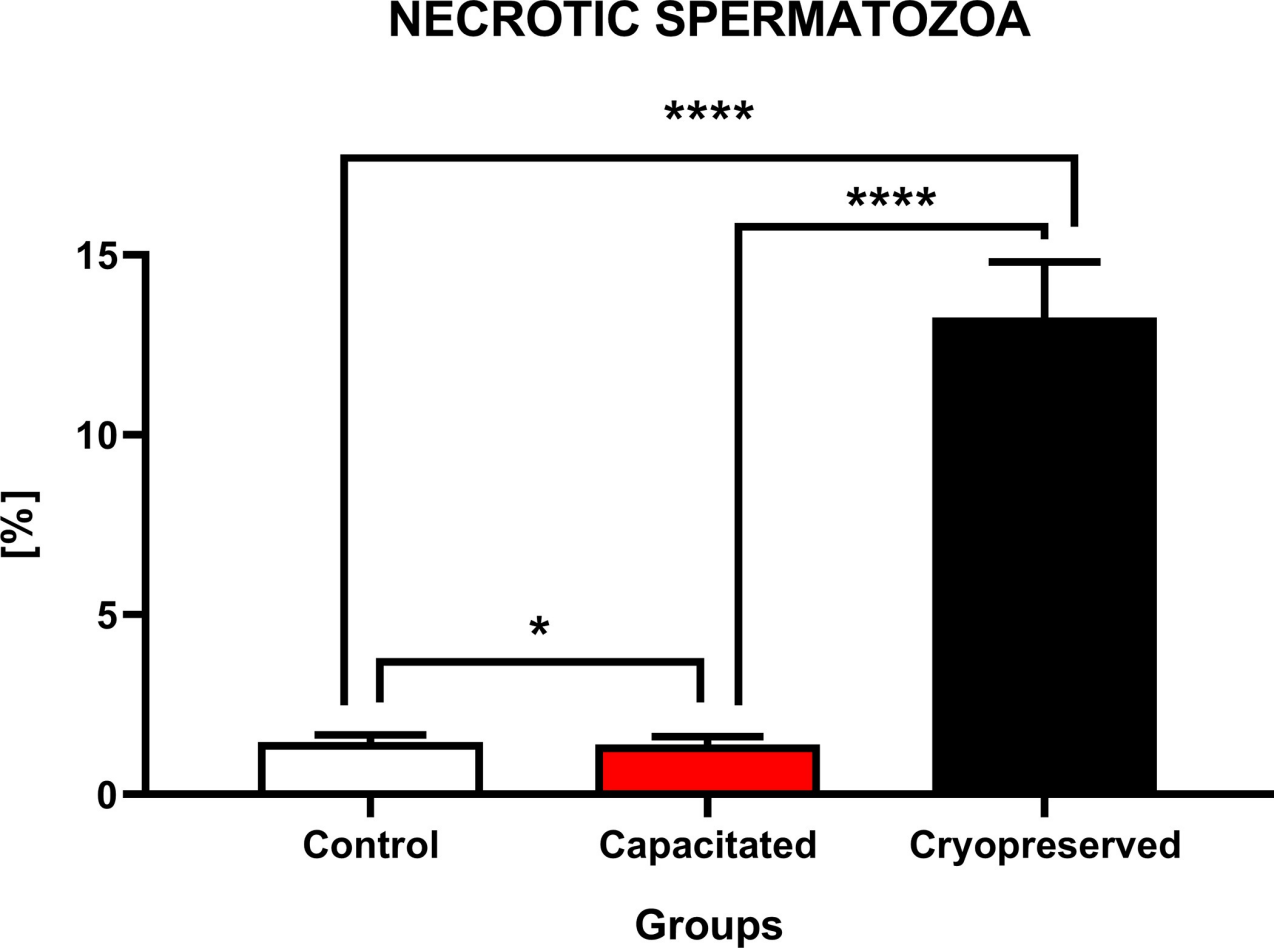
Occurrence of necrotic spermatozoa. The graph showed the level of cell necrosis (%) between the fractions of bull spermatozoa (±SD). The level of significance was set at *P<0.05; **P<0.01; ***P<0.001; ****P<0.0001.

Higher quantity of necrotic cells (Fig 7) was detected in the CRYO group (13.3±1.5%) when compared to CAP (1.4±0.2%) and CTRL (1.5±0.2%). The results indicate a statistical increase (P<0.0001) of necrotic cells in CRYO group against the rest of the groups. A significant difference (P<0.05) in the number of necrotic cells was also visible between CAP and CTRL.

#### DNA fragmentation

**Fig 8:**
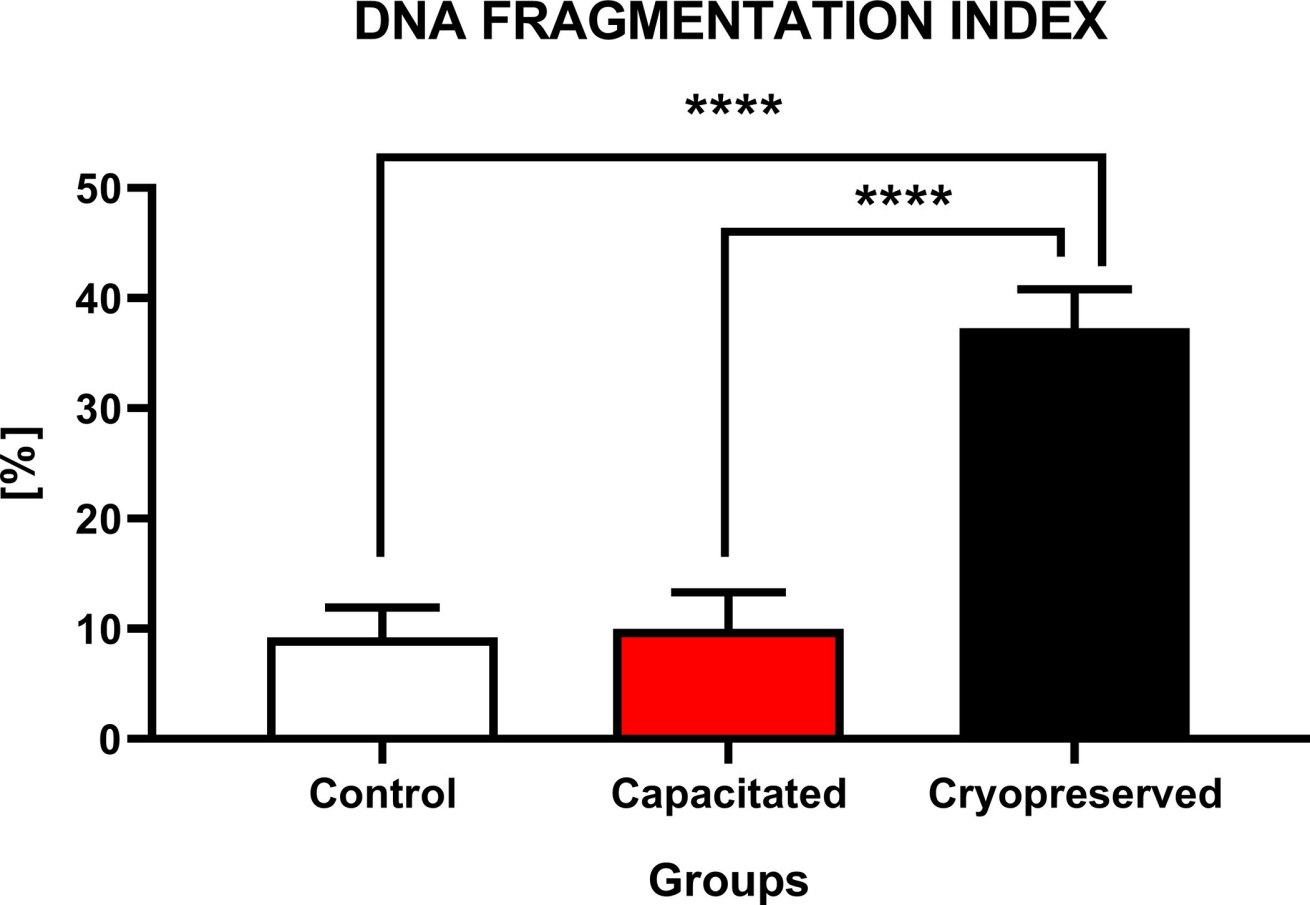
DNA fragmentation index of non-capacitated, *in vitro* capacitated and cryopreserved bovine spermatozoa. The values in the columns represented the percentage (%) of spermatozoa with fragmented DNA between the groups (±SD). The level of significance was set at *P<0.05; **P<0.01; ***P<0.001; ****P<0.0001.

The level of DNA fragmentation (Fig 8) points out to a significant increase (P<0.0001) of fragmented DNA in the CRYO group (37.3±3.5%) against CTRL (9.2±0.1%) and CAP (10±1.2%). No significant changes were observed between CAP and CTRL.

### Capacitation changes

#### Capacitation patterns

**Fig 9:**
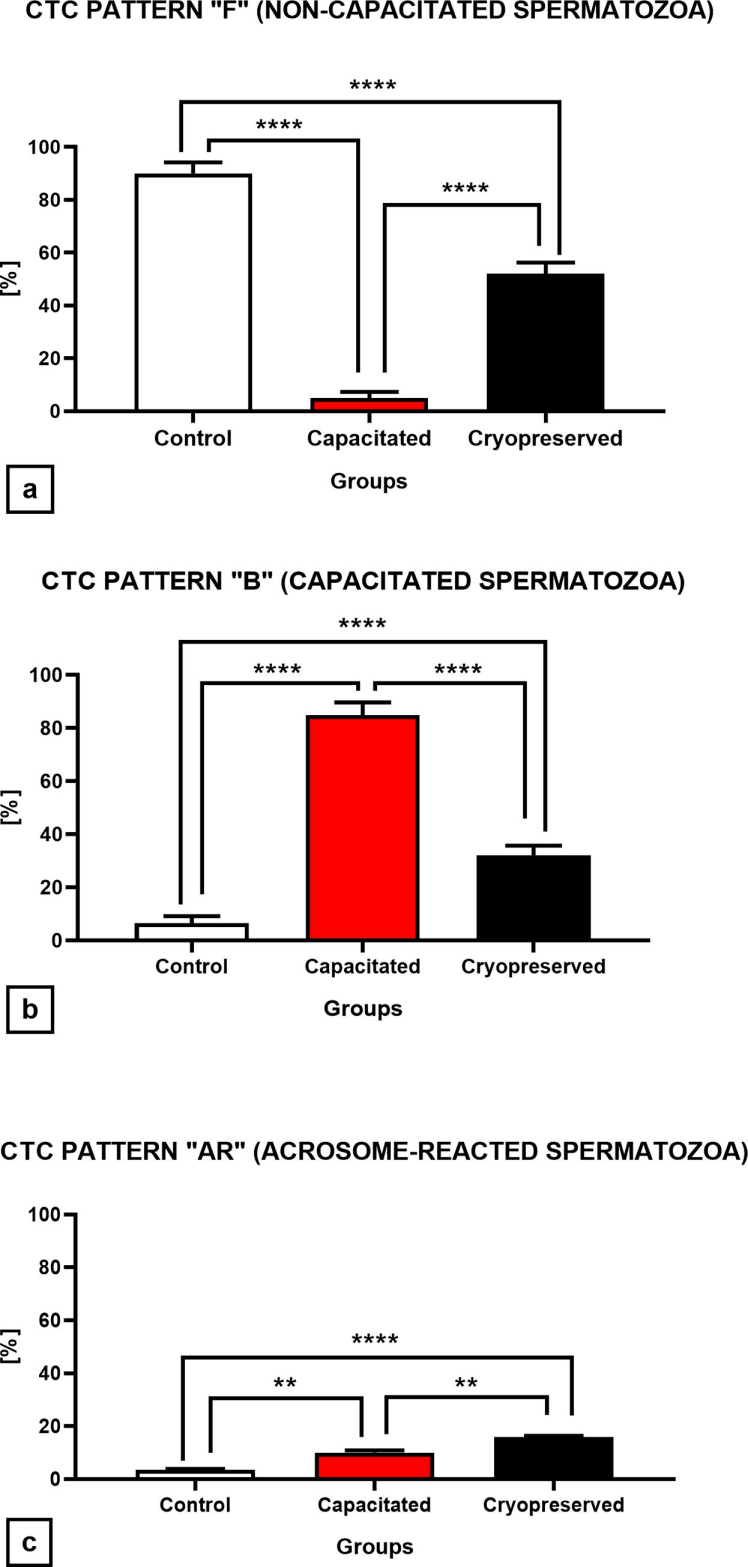
Capacitation CTC patterns of non-capacitated, *in vitro* capacitated and cryopreserved bovine spermatozoa. All graphs showed individual CTC patterns between groups (±SD). (**a**) CTC pattern “F”, non-capacitated spermatozoa. (**b**) CTC pattern “B”, capacitated spermatozoa. (**c**) CTC pattern “AR”, acrosome-reacted spermatozoa. The level of significance was set at *P<0.05; **P<0.01; ***P<0.001; ****P<0.0001.

Based on our microscopic observations (Fig 9A), the highest proportion (90±4.2%) of uncapacitated sperm cells (CTC pattern „F“) was found in the CTRL, with a significance rate of P<0.0001 compared to the CAP and CRYO group. In the CAP group the results point out to a significant decrease (P<0.0001) of uncapacitated cells against CRYO and CTRL. A significant increase (P<0.0001) in the percentage of uncapacitated spermatozoa (52.0±4.3%) was visible in the CRYO group when compared to CAP (5.0±0.4%), however in comparison with the CTRL a significant decrease (P<0.0001) in the proportion of uncapacitated cells was observed. In terms of quantity of cells that underwent capacitation (CTC pattern „B“), the results indicate that the highest proportion (P<0.0001) of capacitated cells (8.05±4.8%) was present in the CAP experimental group. However, a significant increase (P<0.0001) in capacitated cells was also observed in the CRYO group (33.0±0.7%) in comparison with the CTRL (6.5±0.5%) (Fig 9B). The highest proportion of cells which underwent acrosome reaction (CTC pattern „AR“) was observed in the CRYO group (15.9±2.5%) in comparison to CAP (11.0±0.8%) and CTRL (3.6±0.3%). There was a significant increase (P<0.01; P<0.0001) of acrosome-reacted cells in the CRYO group in comparison to the CAP and CTRL group (Fig 9C).

### Oxidative profile

#### Concentration of reactive oxygen species (ROS)

**Fig 10:**
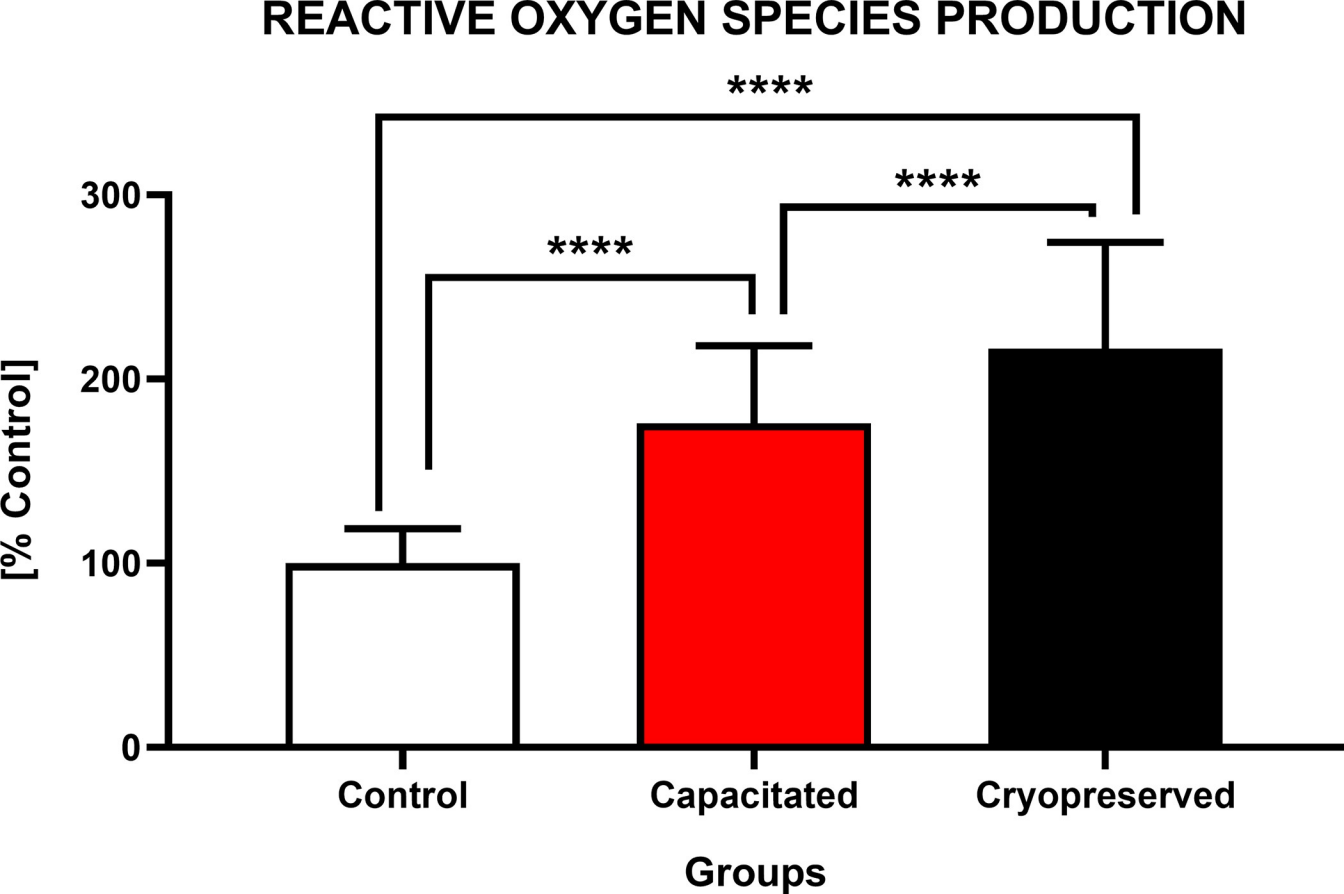
Intracellular ROS production of non-capacitated, *in vitro* capacitated and cryopreserved bovine spermatozoa. Each bar represented the concentration of ROS (%) between the groups (±SD). The level of significance was set at *P<0.05; **P<0.01; ***P<0.001; ****P<0.0001.

Analysis of the ROS generation (Fig 10) revealed a significant increase (P<0.0001) of ROS concentration in CRYO (216.7±6.8%) against CAP (176.2±4.9%) and CTRL (100.0±2.2%). In the case of the CAP group there was a significant decrease (P<0.0001) in the ROS production when compared to the CRYO group and an increase (P<0.0001) in the ROS concentration between CAP and CTRL.

#### Concentration of superoxide radical

**Fig 11:**
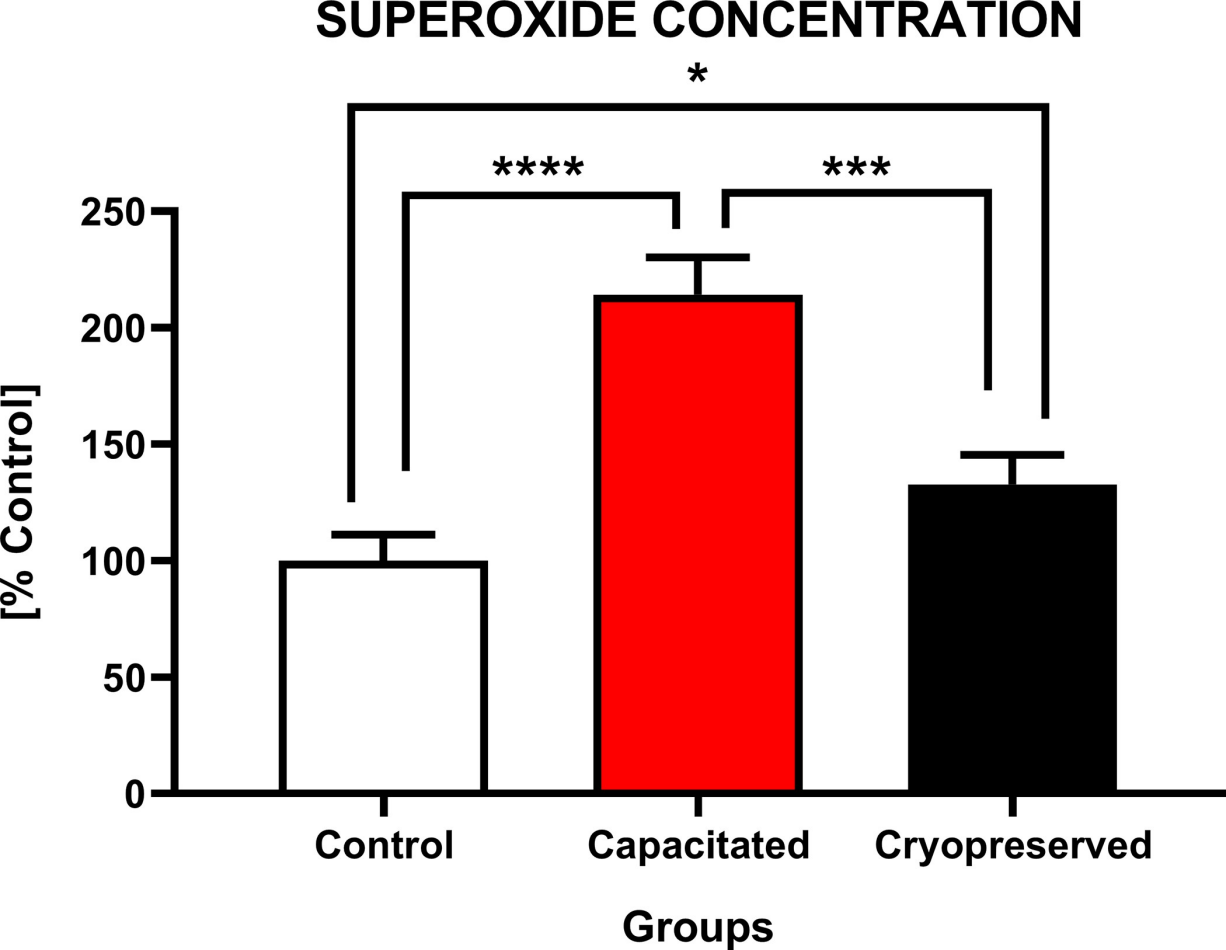
Intracellular superoxide production of non-capacitated, *in vitro* capacitated and cryopreserved bovine spermatozoa. The values in columns showed the concentration (%) of superoxide radical between the groups (±SD). The level of significance was set at *P<0.05; **P<0.01; ***P<0.001; ****P<0.0001.

In contrast with the ROS production, an opposite phenomenon in the superoxide production (Fig 11) was detected. The graph indicates that the highest concentration of superoxide was found in the CAP group (214.1±16.1%). In comparison with the CTRL (100.0±11.2%) and CRYO group (132.7±12.8%) there was a statistical decrease (P<0.001; P<0.0001) in the superoxide production. A statistical increase (P<0.05) in the superoxide concentration was also visible between the CRYO and CTRL group.

#### Concentration of hydrogen peroxide

**Fig 12:**
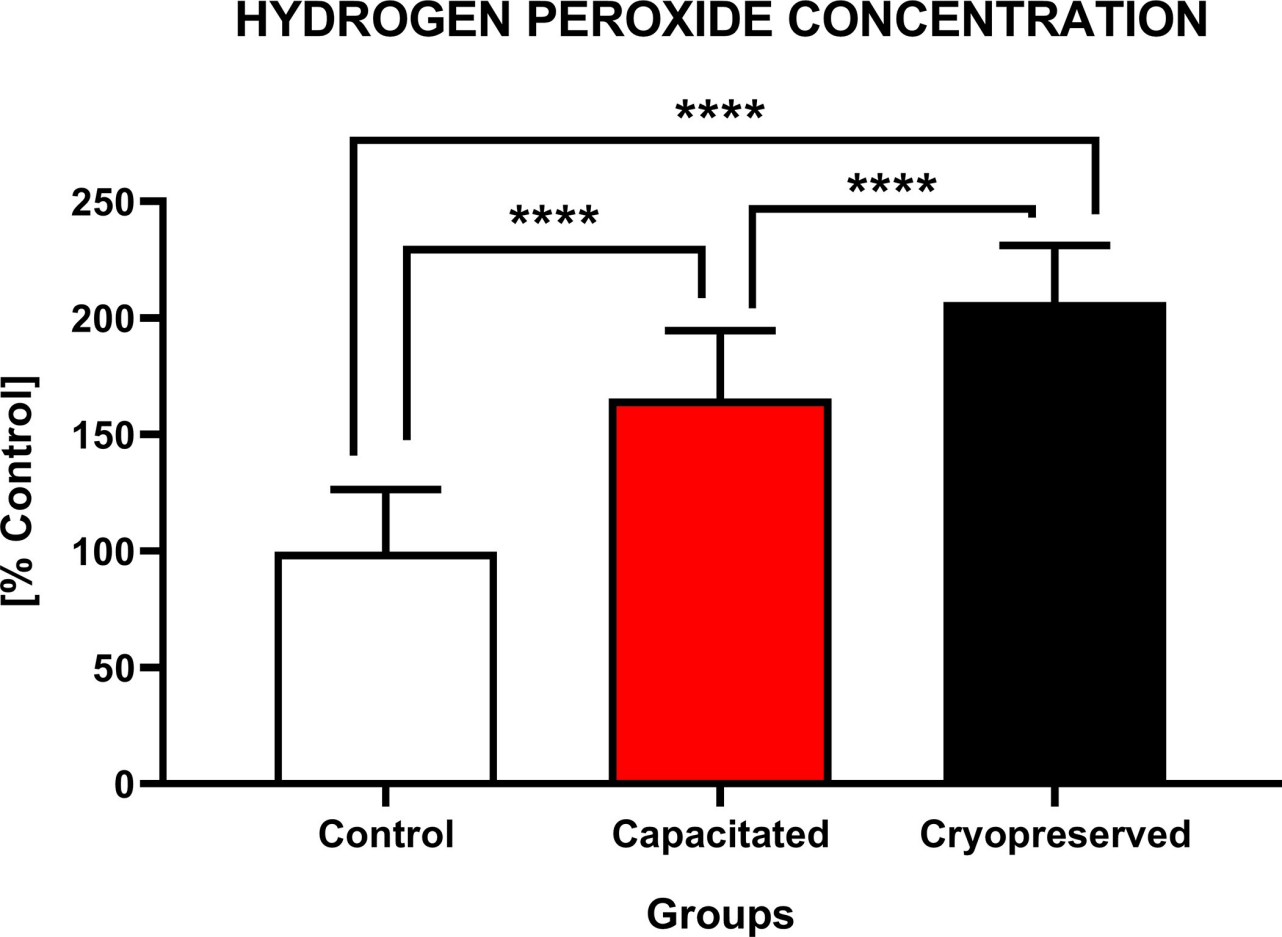
Intracellular production of hydrogen peroxide of non-capacitated, *in vitro* capacitated and cryopreserved bovine spermatozoa. The graph represented the concentration (%) of hydrogen peroxide in each group (±SD). The level of significance was set at *P<0.05; **P<0.01; ***P<0.001; ****P<0.0001.

In the case of hydrogen peroxide concentration, a statistically higher (P<0.0001) concentration was observed in the CRYO group (206.7±4.9%) in comparison to CAP (165.4±5.4%) and CTRL (100.0±4.9%). A significant (P<0.0001) increase in the hydrogen peroxide was visible also between the CAP and CTRL group (Fig 12).

#### Concentration of hydroxyl radical

**Fig 13:**
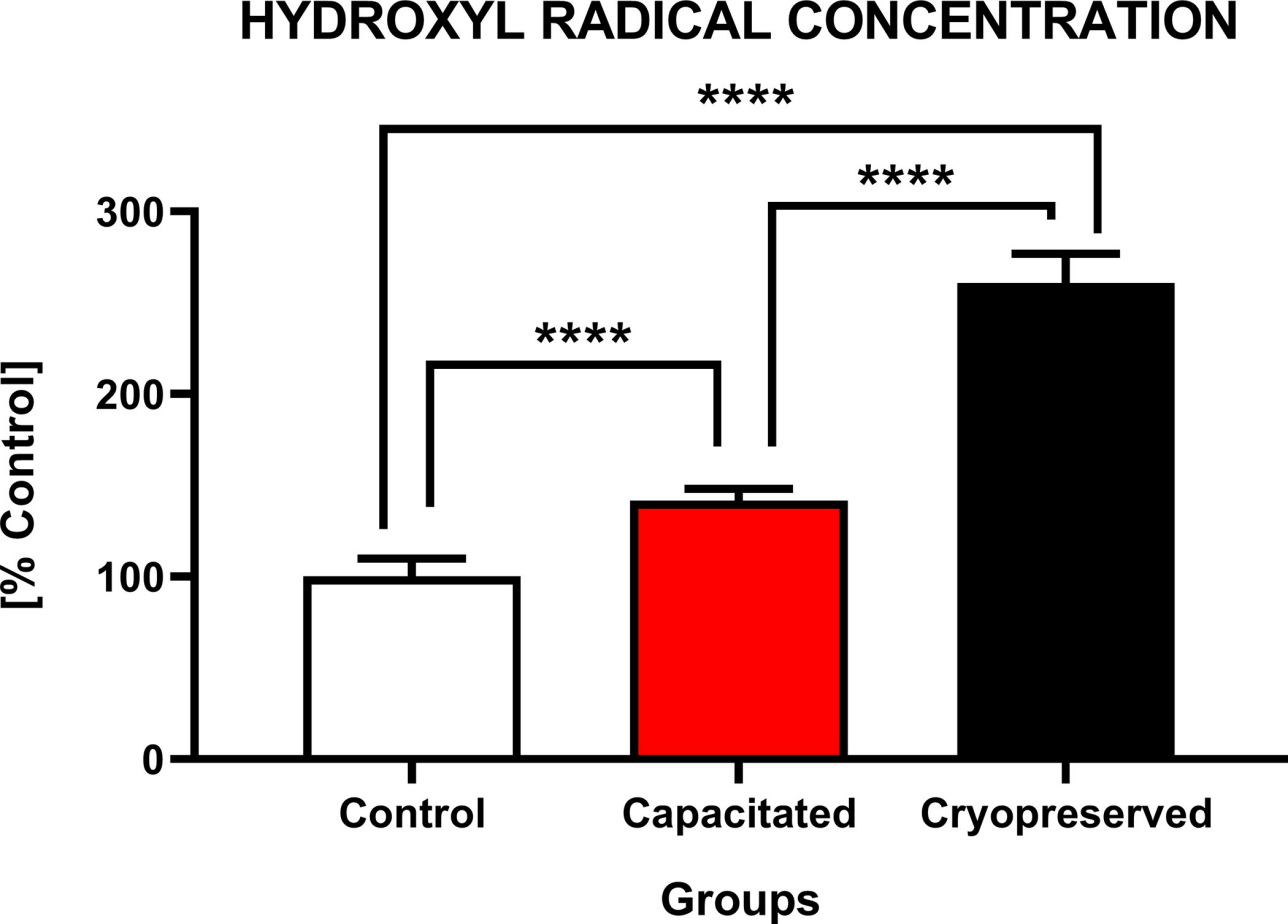
Intracellular production of hydroxyl radical of non-capacitated, *in vitro* capacitated and cryopreserved bovine spermatozoa. Each column presents the concentration (%) of hydroxyl radical by comparing all groups with each other (±SD). The level of significance was set at *P<0.05; **P<0.01; ***P<0.001; ****P<0.0001.

Similarly, to the ROS and hydrogen peroxide generation, the highest concentration of hydroxyl radical (Fig 13) was detected in the CRYO group (260.9±16%). A significantly increased (P<0.0001) concentration of hydroxyl radical was observed in both experimental groups (CRYO and CAP) against CTRL (100.0±9.8%). The difference was characterized by a dramatic increase (P<0.0001) in the production of hydroxyl radical between CAP (141.7±6.3%) and CRYO.

## Discussion

The present study provides a more detailed look on the impact of cryopreservation on a possible premature activation of capacitation, also known as cryocapacitation on cryopreserved bovine spermatozoa. The collected data may also help to identify the connection between cryodamage and premature capacitation of spermatozoa by comparing their qualitative as well as quantitative parameters under *in vitro* conditions. While a number of previous studied have indicated changes thar occur during a natural and/or cryo-induced capacitation [4,5,8], none of these has taken into account a wide variety of traditional and non-conventional markers accounting for the major structural and functional properties of the male gamete. Moreover, while it is known that free radicals play a pivotal role in the process of physiological and cryo-induced capacitation, there are no data yet distinguishing among the major ROS types and their fluctuations during both processes. This information may help us to understand which ROS concentrations may be considered physiological or pathological, and thus may be of assistance when designing new antioxidant supplements that may help to stabilize ROS within tolerable levels during and after cryopreservation.

Motility is considered as a primary quality factor of ejaculates and is essential for the fertilization ability of sperm cells. However, the analysis of the kinetics of sperm movement through the CASA system provides us with only initial data. Our results confirm that the motility of cryopreserved samples decreased almost by 20%. These findings corelate with previous studies, which revealed that due to cryopreservation, the motion of bovine spermatozoa decreases almost by 50% when compared to fresh samples [19–21].

Moreover, it was confirmed that the egg yolk, a common component of cryopreservation media has the ability to interact with glycoprotein-60 and phospholipase A_2_, found in the bulbourethral secretion, and exhibits triacylglycerol hydrolase activity that decreases the sperm motility by disruption of the cell membrane. The hydrolysis of egg yolk is catalyzed by phospholipase A_2_, which leads to the formation of fatty acids and lysophophatidylcholine (LPC). These interactions are responsible for the stimulation of cryocapacitation, acrosomal damage and the loss of motility or viability of spermatozoa [22].

A variety of animal-based extenders have been developed through the years for the improvement of post-thaw quality of semen samples. In general, egg yolk extenders contain low density lipoproteins (LDL), which can protect spermatozoa from cryodamage by formation of a protective barrier all around the cell surface as a cryoprotective fraction of yolk or by replacing damaged and lost phospholipids due to cryopreservation. The protective mechanism depends on a mutual interactation between LDL with the family of lipid binding proteins in bull seminal plasma. On the other hand, high density lipoproteins (HDL) and minerals can inhibit the sperm cell respiration and motility [23,24]. Pillet et al. suggested that application of only egg yolk plasma fraction could be a solution to reduce a potentially negative impact of HDL since the plasma fraction contains mostly LDL (85%), which includes triglycerides, cholesterol, apoproteins, phospholipids and 15% of livetins [25]. Another commonly used animal-based cryoprotectant or supplement for glycerol-free trehalose or egg yolk-based extenders is skim milk. Skim powdered milk is a non-permeable cryoprotectant, which presents with the ability to protect the sperm tail, reduce sperm agglutination and preserve a normal morphology during cryopreservation and after thawing. [26,27]. Casein micelles are considered to be a major protective constituent of skim milk. Similarly to egg yolk, milk fat globules contain choline phospholipids, which are able to bond with seminal plasma proteins [28]. From the perspective of *in vitro* fertility of post-thaw semen used for insemination, Üstüner et al. observed that the highest cleavage and blastocyst formation was in the group treated with 20% of egg yolk when compared to different concentrations of soybean-derived lecithin [29]. In contrary, the research team of Aires et al. confirmed a significantly higher (P<0.0001) insemination success in soya lecithin extended bovine spermatozoa (70.45%) in comparison to TRIS-egg yolk extender (67.85%), which can be used as an alternative diluent [30]. Skim milk or Tris-glucose are the most often used extenders for buck and ram semen preservation. However, the protein fraction of bulbourethal gland secretion in seminal plasma can be damaged following dilution in egg yolk or milk-based extender. This is the reason why these milk-based extenders are often enriched with a variety of supplements such as hyaluronic acid, L-arginine or cholesterol-loaded cyclodextrin [31–33]. Dorado et al. examined the differences between goat semen doses cryopreserved in milk-based extender vs. TRIS extender, where intra-cervical insemination and higher pregnancy rates were observed in the milk extender (52.4%) against TRIS (42.9%) [34]. Interestingly, in the case of ram spermatozoa TRIS extender showed a better efficiency in the case of sperm motility, membrane integrity as well as an increased number of un-capacitated spermatozoa. However, ram semen diluted in milk-based extender exhibited a more favourable fertility range after vaginal insemination of sheep’s and hence was recommended as a diluent [35].

The motility rate is closely related to the course of capacitation since spermatozoa undergo a process called hyperactivation. This phenomenon is microscopically observable, and it is characterized by a rapid oscillation of the sperm flagellum. In terms of *in vitro* activation of capacitation, we observed an almost 10% increase in the proportion of motile sperm when compared to the control. Evaluation of *in vitro* capacitated cells suggests a significant increase (P<0.05) of hyperactivated bovine spermatozoa following exposure to the capacitation medium against the control [36]. Hyperactivated motility of mammalian spermatozoa is a necessary step prior to capacitation, which is directly affected by the concentration of cAMP and intracellular calcium. During capacitation, numerous sperm proteins undergo phosphorylation, which is regulated by the cAMP pathway and protein kinase A activation (PKA) [37].

The level of sperm motility is directly proportional to the metabolic activity of mitochondria in the cell, which are located in the middle section of flagellum and provide energy in the form of adenosine triphosphate (ATP) for flagellar movement of spermatozoa. Cryopreservation increases the risk of the loss of mitochondrial membrane potential and a decrease of mitochondrial respiration, leading to a reduction in the ATP synthesis and a decreased mitochondrial ROS production, which was previously confirmed by the sublethal effect of mitochondrial permeability transition to sperm metabolism [38]. Unlike in the *in vitro* capacitated spermatozoa, the cryopreserved group suffered from mechanical cryoinjury caused by the presence of ice crystals, which could have led to a swelling of the mitochondrial matrix and a disruption of mitochondrial membranes.

Low temperatures induced a significant increase in the proportion of bovine spermatozoa with a low mitochondrial potential and a decrease of the mitochondrial activity in thawed samples when compared to fresh semen [39,40]. During cryopreservation the axonemal proteins (dyneins, tubulins and heat shock proteins) as well as the mitochondrial proteins (succinate dehydrogenase and ATP synthase) undergo lipid peroxidation, which leads to a disruption of the cytoskeleton structure and causes adverse effects on the electron transport chain. As this chain is an essential part of the mitochondrial oxidative phosphorylation, due to its disruption, ATP depletion and spontaneous generation of ROS occur, which may eventually initiate the apoptotic cascade [41]. Mitochondria may subsequently change their morphology by increasing their volume and wrinkling the membrane. Under *in vitro* conditions, mitochondrial respiratory activity increases in the presence of the oxidizable substrates in the capacitation medium, with an increase in oxygen consumption. Due to high concentration of ATP produced by glucose oxidation, adenylyl cyclase activity is thus stimulated in the presence of bicarbonate (HCO_3_^-^), leading to an increased cAMP stimulation and PKA activation [42].

Signal transduction, which promotes intracellular cAMP synthesis is activated by capacitation. cAMP forms a complex with protein kinase A in the presence of high concentrations of Ca^2+^, leading to hyperpolarization of the plasma membrane of cells, hyperactivation, capacitation and acrosome reaction. It has been reported that the synthesis of intracellular Ca^2+^ is induced by cryopreservation after thawing, which leads to premature capacitation and acrosome response [43,44]. Extracellular cAMP induces a significant increase in the proportion of hyperactivated sperm cells, which correlated with the mitochondrial respiratory activity detected by the lipophilic cationic probe. As mentioned before, cAMP modulates PKA activity, sperm hyperactivation, and tyrosine phosphorylation, which is characteristic for capacitated spermatozoa. The molecule is also responsible for an increased AMP/ATP ratio, with PKA/PKC activity increasing the regulation of mitochondrial membrane potential via AMPK (5-adenosinemonophospate-activated protein kinase), supporting the theory that cAMP has a role as an autocrine/paracrine messenger of capacitation [45]. On the other hand, due to the cryopreservation process, the activity of PKA is inhibited together with AMPK. The activity of these kinases is reflected in the proportion of motile sperm after thawing. It means that the inhibition of AMPK activity after thawing is associated with a decrease in extracellular cAMP concentration and may be responsible for the loss of energy required for the sperm movement, which is characterized by a decreased motility. If mitochondrial respiratory activity decreases, it will affect the entire cascade of capacitation [46].

One of the primary issues associated with sperm cryopreservation is a significant loss of viability, particularly following thawing. A decreased cell viability is a result of cryodamage, leading to the disintegration of phospholipid structures and collapse of the consolidation architecture of the plasmatic membrane [47]. The loss of membrane integrity in frozen-thawed bovine spermatozoa was confirmed in studies, where a decline of cell viability was observed in the range of 20–30% [40,48].

We may hypothesize that membrane alternations during freezing are associated with changes in the asymmetry of the phospholipid bilayer which negatively affects the functional state of the membrane. Frozen-thawed spermatozoa are able to omit a traditional capacitation cascade through a massive degradation of cholesterol from the plasmatic membrane [49,50].

Lipids and proteins that are in the liquid state become solidified into gel because of low temperatures during the cryopreservation process. Furthermore, spermatozoa are exposed to a hyperosmotic environment that induces the uptake of water and ions across the membrane, leading to cell dehydration. This phenomenon is accompanied by a typical swelling and coiling of the sperm flagellum and formation of intracellular ice crystals [51].

Like the plasma membrane, the mitochondrial membrane undergoes cryogenic damage due to ice crystal formation, which would explain the positive correlation between the mitochondrial membrane potential and membrane integrity, given a similar lipid-protein composition of these membranes. In the case of mitochondrial membrane damage, ions, and ROS escape from mitochondria, reducing the electrical potential and increasing the risk of prooxidative damage to membrane lipids and DNA. A decrease in the mitochondrial potential and increased cytochrome c oxidase oxidation is characteristic of a partial rupture of cellular as well as mitochondrial membranes. This structural damage of membranes is a possible explanation for a partial loss of motility after cryopreservation [52].

The presence of an intact acrosome (containing hydrolytic enzymes) is a prerequisite for a successful course of fertilization. Cryopreservation causes a decrease in the percentage of viable cells and an increase in the proportion of spermatozoa with an activated or damaged acrosome. It was reported that the integrity of the acrosome decreases due to thawing, the acrosomal cap is thought to be damaged, reducing the fertilization potential of spermatozoa in bulls [53], boars [54] and rams [55]. After thawing a formation of blistering structures in the anterior region of the acrosome has been previously reported. Premature release of acrosomal enzymes such as hyaluronidase and acrosin reduce the quality of ejaculate after thawing. Another contraindication is the thermolability of cytoskeletal proteins (actin, F-actin and β-dystrobverin), leading to their eventual depolymerization and change in their distribution [20,56].

Previous studies reported that the rate of cryopreserved bovine spermatozoa with acrosomal damage increased up to 10–20% when compared to fresh samples [21,57].

Another complication caused by cryodamage is cell necrosis, which is most often connected with heat shock and the presence of intra or extra cellular ice crystals. The main problem lies in the transition of cells from -130°C to -15°C, which they have to go through twice, once during freezing and later by thawing. A characteristic manifestation of cell death due to necrosis is loss of cell membrane integrity [58]. In general, the presence of necrotic cells after thawing is not so dramatic but a high proportion of cells have sublethal damage due to cryodamage. These alternations include DNA fragmentation, loss of plasma membrane integrity, chromatin condensation, caspase activation and loss of mitochondrial membrane potential or opening of mitochondrial permeability transition (MPT) pores, which impact the cytosolic release and activation of proapoptotic factors. Oxidative stress and Ca^2+^ levels have been shown to be most important inducers of MPT pores. This theory suggests the involvement of these pores in the initiation of apoptotic or necrotic cell changes [39,59,60].

The level of DNA fragmentation determines a successful course of fertilization. The presence of dead, damaged or abnormal cells in cryopreserved samples could be potential source of ROS and increase the risk of oxidative injury. Cryopreservation procedure itself increases the proportion of damaged spermatozoa and leads into a reduction of DNA integrity in post-thawed samples. Moreover, cryopreservation can disintegrate chromatin and increase the level of DNA denaturation [61,62]. Freezing and thawing procedures affect DNA integrity, which is responsible for molecular and epigenetic modifications. As mentioned before, DNA fragmentation is related to cryopreservation, which lead to double strand breaks caused by high concentration of ROS, malfunction of DNA repair enzymes and mechanical stress of cells characterized by chromatin compaction. These DNA changes are usually positively correlated with level of apoptotic cells in cryopreserved samples of bulls [63].

In terms of the capacitation status, we were able to identify the CTC patterns and quantified the number of capacitated and uncapacitated cells as well as cells which underwent acrosome reaction. Previous studies indicate a statistical increase of capacitated cells („B”pattern) as well as acrosome reacted spermatozoa („AR”pattern) and suggest that cryopreservation procedure induces capacitation-like changes in cryopreserved bovine spermatozoa, which corroborate with our research [57,64].

It is well known that a deficiency of antioxidant activity during cryopreservation and degradation of L-amino acids of damaged or dead cells contribute significantly to an increased generation of ROS. Excessive ROS production destabilizes sperm cell membranes due to disruption of disulphide bonds of membrane proteins, peroxidation of membrane phospholipids, and modification of the sperm glycocalyx [41,65]. Overproduction of ROS initiates oxidative damage to membrane phospholipids, which leads to the formation of toxic products such as malondialdehyde (MDA) and acrolein. Because of oxidative damage, some molecules of cholesterol, phosphatidylethanol amine and phosphatidylcholine are released from the membrane, reducing the membrane integrity and the loss of semipermeable properties [66]. Several studies confirm that spermatozoa are capable to generate ROS via the redox regulation of tyrosine phosphorylation as observed in the buffalo [67] or bull [68]. These biochemical reactions involve a stimulation of cAMP generation and inhibition of tyrosine phosphatase activity [69]. Furthermore, the antioxidant capacity of seminal plasma, which provides enzymatic and non-enzymatic proteins is reduced due to dilution of semen with cryoaditives [70].

As such, we may conclude that the dramatic increase in the concentration of free radicals in the cryopreserved group is associated with a decrease in membrane integrity and loss of antioxidant components of the cytoplasm. However, a certain concentration of free radicals is essential for sperm hyperactivation and capacitation. Production of ROS is an accompanying manifestation of an early activation of sperm capacitation. The main free radicals produced by sperm cells include superoxide anion (O_2_•^-^), peroxynitrite (ONOO-) and nitrogen dioxide (NO_2_•), which is subsequently dismuted to hydrogen peroxide (H_2_O_2_). The presence of membrane NADPH oxidase is thought to be responsible for the production of O_2_•^-^ [71]. The physiological role of O_2_•^-^ was demonstrated by heparin-induced capacitation of cryopreserved bovine spermatozoa. During the incubation, there was a 3-fold increase in oxygen consumption and a 5-fold increase in the superoxide production by sperm cells. This observation supports the hypothesis that the induction of capacitation leads to activation of mitochondrial respiration due to an increased energy requirement and O_2_•^-^ production by membrane NADPH oxidase [72].

We may speculate that the presence of exogenous superoxide could induce capacitation-like changes and acrosome reaction in cryopreserved bovine spermatozoa. Higher concentration of superoxide in the capacitated group explains that this free radical may play a primary role in terms of sperm hyperactivation and capacitation.

The reduction of O_2_•^-^ in the cryopreserved group could be explained as the result of his dismutation into hydrogen peroxide, which could be decomposed in the presence of ferrous ions into hydroxyl radical [73].

This theory is supported by the fact that a decline in the concentration of superoxide and increased concentration of hydrogen peroxide and hydroxyl radical was observed in the cryopreserved group. It may be explained by a premature consumption of a superoxide by capacitation-like changes induced by low temperatures.

Due to a decreased activity of antioxidant enzymes such as glutathione peroxidase and catalase during cryopreservation the degradation of hydrogen peroxide into oxygen and water is insufficient. On the other hand, an increased activity of superoxide dismutase catalyses the dismutation of the superoxide anion to hydrogen peroxide, leading to its accumulation leading to the development of oxidative damage and initialization of the lipid peroxidation by the production of hydroxyl radical. In addition, the synergic action of hydrogen peroxide and peroxynitrite has been shown to be responsible for controlling oxidative processes and regulating the sperm capacitation [69,74].

## Conclusions

The presented study offers a detailed view into sperm cryodamage and summarizes the consequences of low temperatures on the functional activity, structural integrity, course of capacitation and oxidative profile of male gametes. Our results indicate that the cryopreservation process negatively affected almost all selected parameters, which may lead to a substantial reduction of the semen quality and an undesirable possible activation of premature capacitation as a by-product of cryodamage. Cryopreserved spermatozoa exhibited obvious signs of membrane as well as acrosomal damage, loss of movement, cell necrosis and excessive production of free radicals except for superoxide. Our findings could be applied in future experiments in the field of animal andrology and provide a new view to the molecular processes affected by cryopreservation including cryocapacitation.

## Supporting information

S1 File Summarization of the volume, sperm concentration, volume of diluents and final concentration of bovine spermatozoa in each sample. (DOCX)
