# Supplementary material for: In vitro versus cryo-induced capacitation of bovine spermatozoa, part 1: Structural, functional, and oxidative similarities and differences
Source: PLoS One. 2022 Oct 21;17(10):e0276683. doi: 10.1371/journal.pone.0276683 (PMC9586399; doi:10.1371/journal.pone.0276683)
Supplement: S1 File — (DOCX) [file pone.0276683.s001.docx]

**S1 Table. Summarization of the volume, sperm concentration, volume of diluents and final concentration of bovine spermatozoa in each sample**

| **Sample number** | **Volume** | **Concentration** | **Total count** | **Sample volume used for each group** | **Volume of medium for the CTR and CAP group** | **Final sperm concentration in the CTR and CAP group** | **Volume of medium for the CRYO group** | **Final sperm concentration in the CRYO group** |
| --- | --- | --- | --- | --- | --- | --- | --- | --- |
| 1 | 7.00 mL | 1 907.00 x10^6^/mL | 13 348.90 x10^6^ | 2.00 mL | 80.00 mL | 47.70 x10^6^/mL | 86.70 mL | 44.00 x10^6^/mL |
| 2 | 4.50 mL | 2 501.40 x10^6^/mL | 11 256.30 x10^6^ | 1.50 mL | 60.00 mL | 62.50 x10^6^/mL | 85.30 mL | 44.00 x10^6^/mL |
| 3 | 7.50 mL | 1 972.90 x10^6^/mL | 14 796.80 x10^6^ | 2.50 mL | 100.00 mL | 49.40 x10^6^/mL | 112.10 mL | 44.00 x10^6^/mL |
| 4 | 9.50 mL | 1 663.10 x10^6^/mL | 15 799.50 x10^6^ | 3.00 mL | 120.00 mL | 41.60 x10^6^/mL | 113.40 mL | 44.00 x10^6^/mL |
| 5 | 4.50 mL | 1 432.80 x10^6^/mL | 6 447.60 x10^6^ | 1.50 mL | 60.00 mL | 35.90 x10^6^/mL | 48.90 mL | 44.00 x10^6^/mL |
| 6 | 7.00 mL | 2 062.40 x10^6^/mL | 14 136.80 x10^6^ | 2.00 mL | 80.00 mL | 51.60 x10^6^/mL | 93.80 mL | 44.00 x10^6^/mL |
| 7 | 7.50 mL | 1 863.40 x10^6^/mL | 13 975.50 x10^6^ | 2.50 mL | 100.00 mL | 46.60 x10^6^/mL | 105.90 mL | 44.00 x10^6^/mL |
| 8 | 8.00 mL | 1 985.30 x10^6^/mL | 15 882.10 x10^6^ | 2.50 mL | 100.00 mL | 49.70 x10^6^/mL | 112.80 mL | 44.00 x10^6^/mL |
| 9 | 8.00 mL | 2 029.80 x10^6^/mL | 16 238.20 x10^6^ | 2.50 mL | 100.00 mL | 50.80 x10^6^/mL | 115.30 mL | 44.00 x10^6^/mL |
| 10 | 7.00 mL | 2 000.10 x10^6^/mL | 14 000.70 x10^6^ | 2.00 mL | 80.00 mL | 50.00 x10^6^/mL | 90.90 mL | 44.00 x10^6^/mL |
| 11 | 9.50 mL | 1 636.60 x10^6^/mL | 15 547.70 x10^6^ | 3.00 mL | 120.00 mL | 40.90 x10^6^/mL | 111.60 mL | 44.00 x10^6^/mL |
| 12 | 8.50 mL | 1 720.60 x10^6^/mL | 14 625.10 x10^6^ | 2.50 mL | 100.00 mL | 43.00 x10^6^/mL | 97.80 mL | 44.00 x10^6^/mL |
| 13 | 5.00 mL | 1 893.70 x10^6^/mL | 9 468.50 x10^6^ | 1.50 mL | 60.00 mL | 47.40 x10^6^/mL | 64.60 mL | 44.00 x10^6^/mL |
| 14 | 6.50 mL | 2 066.10 x10^6^/mL | 13 429.70 x10^6^ | 2.00 mL | 80.00 mL | 51.70 x10^6^/mL | 93.90 mL | 44.00 x10^6^/mL |
| 15 | 9.00 mL | 1 717.30 x10^6^/mL | 11 162.50 x10^6^ | 3.00 mL | 120.00 mL | 42.90 x10^6^/mL | 117.00 mL | 44.00 x10^6^/mL |
| 16 | 7.50 mL | 1 746.20 x10^6^/mL | 13 096.50 x10^6^ | 2.50 mL | 100.00 mL | 43.70 x10^6^/mL | 99.20 mL | 44.00 x10^6^/mL |
| 17 | 7.50 mL | 1 923.80 x10^6^/mL | 14 428.50 x10^6^ | 2.50 mL | 100.00 mL | 48.10 x10^6^/mL | 109.40 mL | 44.00 x10^6^/mL |
| 18 | 8.00 mL | 1 709.90 x10^6^/mL | 13 679.20 x10^6^ | 2.50 mL | 100.00 mL | 42.80 x10^6^/mL | 97.20 mL | 44.00 x10^6^/mL |
| 19 | 6.00 mL | 1 630.70 x10^6^/mL | 9 784.20 x10^6^ | 2.00 mL | 80.00 mL | 40.80 x10^6^/mL | 74.20 mL | 44.00 x10^6^/mL |
| 20 | 8.00 mL | 1 826.90 x10^6^/mL | 14 615.00 x10^6^ | 2.50 mL | 100.00 mL | 45.70 x10^6^/mL | 103.80 mL | 44.00 x10^6^/mL |
| 21 | 9.50 mL | 1 925.90 x10^6^/mL | 18 296.00 x10^6^ | 3.00 mL | 120.00 mL | 48.10 x10^6^/mL | 131.40 mL | 44.00 x10^6^/mL |
| 22 | 8.50 mL | 1 687.60 x10^6^/mL | 14 344.60 x10^6^ | 2.50 mL | 100.00 mL | 42.20 x10^6^/mL | 95.90 mL | 44.00 x10^6^/mL |
| 23 | 7.50 mL | 2 047.60 x10^6^/mL | 15 352.50 x10^6^ | 2.50 mL | 100.00 mL | 51.20 x10^6^/mL | 116.40 mL | 44.00 x10^6^/mL |
| 24 | 8.00 mL | 1 251.50 x10^6^/mL | 10 012.00 x10^6^ | 2.50 mL | 100.00 mL | 31.30 x10^6^/mL | 71.10 mL | 44.00 x10^6^/mL |
| 25 | 8.50 mL | 1 760.20 x10^6^/mL | 14 961.70 x10^6^ | 2.50 mL | 100.00 mL | 44.00 x10^6^/mL | 100.00 mL | 44.00 x10^6^/mL |
| 26 | 8.00 mL | 1 939.80 x10^6^/mL | 15 518.40 x10^6^ | 2.50 mL | 100.00 mL | 48.50 x10^6^/mL | 110.20 mL | 44.00 x10^6^/mL |
| 27 | 7.00 mL | 1 671.80 x10^6^/mL | 11 702.60 x10^6^ | 2.00 mL | 80.00 mL | 41.80 x10^6^/mL | 76.00 mL | 44.00 x10^6^/mL |
| 28 | 5.50 mL | 1 902.30 x10^6^/mL | 10 462.70 x10^6^ | 1.50 mL | 60.00 mL | 47.60 x10^6^/mL | 64.90 mL | 44.00 x10^6^/mL |
| 29 | 9.00 mL | 1 737.80 x10^6^/mL | 15 640.20 x10^6^ | 3.00 mL | 120.00 mL | 43.50 x10^6^/mL | 118.50 mL | 44.00 x10^6^/mL |
| 30 | 8.00 mL | 1 716.00 x10^6^/mL | 13 728.00 x10^6^ | 2.50 mL | 100.00 mL | 42.90 x10^6^/mL | 97.50 mL | 44.00 x10^6^/mL |
